# Complete Genomic Sequence of European Bat Lyssavirus 1, Isolated from Eptesicus isabellinus in Spain

**DOI:** 10.1128/genomeA.01518-14

**Published:** 2015-02-12

**Authors:** Denise A. Marston, Sonia Vázquez-Morón, Richard J. Ellis, Emma L. Wise, Lorraine M. McElhinney, Xavier de Lamballerie, Anthony R. Fooks, Juan E. Echevarría

**Affiliations:** a Wildlife Zoonoses & Vector-Borne Diseases Research Group, Animal and Plant Health Agency (APHA), Surrey, United Kingdom; c Specialist Scientific Support Department, Animal and Plant Health Agency (APHA), Surrey, United Kingdom; b Centro Nacional de Microbiología, Instituto de Salud Carlos III, Majadahonda, Madrid, Spain; d National Consortium for Zoonosis Research, University of Liverpool, Liverpool, United Kingdom; e UMR_D 190,”Emergence de Pathologies Virales,” Aix Marseille University, Marseille, France; f Department of Clinical Infection, Microbiology and Immunology, University of Liverpool, Liverpool, United Kingdom; g Centro de Investigación Biomédica en Red de Epidemiología y Salud Pública (CIBERESP), Madrid, Spain

## Abstract

All members of the lyssavirus genus cause the disease rabies. European bat lyssavirus 1 (EBLV-1) viruses are divided genetically into three groups according to geographic location and host reservoir. We report here the first genome sequence for an EBLV-1 isolated from Eptesiscus isabellinus in the Iberian Peninsula, Spain.

## GENOME ANNOUNCEMENT

There are 14 classified lyssavirus species. In Europe, bat rabies cases are dominated by two lyssavirus species: European bat lyssavirus 1 (EBLV-1) and European bat lyssavirus 2 (EBLV-2) (1). EBLV-1 molecular epidemiological investigations separated the species into 2 sublineages (1a and 1b) (2). Analysis of EBLV-1 viruses from Eptesiscus isabellinus in the Iberian Peninsula has demonstrated that these viruses are genetically similar, yet distinct from other EBLV-1 circulating in Eptesiscus serotinus (3). Full-genome sequences are available for five EBLV-1a and a single EBLV-1b virus. Here, we describe full-genome sequencing of an EBLV-1 virus (292R07, RV2416), isolated from an infected E. isabellinus bat from the Iberian peninsula (Granada), Spain, in 2007.

RNA from mouse brain homogenate (passage 1) was prepared for next-generation sequencing on the MiSeq platform. Briefly, TRIzol extracted viral RNA was depleted of host genomic DNA and rRNA as described previously (4). Double-stranded (ds) cDNA was synthesized from 50 ng RNA by use of a random cDNA synthesis system (Roche), according to the manufacturer’s instructions. The ds cDNA was purified using Ampure XP magnetic beads (Beckman Coulter), and 1 ng was used for the Nextera XT DNA sample preparation kit (Illumina). A sequencing library was prepared according to the manufacturers’ instructions and sequenced on an Illumina MiSeq with 2 × 150-bp paired-end reads following standard Illumina protocols. The total reads (2,032,092) were mapped to a reference sequence (EU293112) using BWA (v0.7.5a-r405) (5) and was visualized in Tablet (6). A modified samtools/vcfutils (7) script was used to generate an intermediate consensus sequence in which any indels relative to the original reference sequence were appropriately called. The intermediate consensus was used as the reference for subsequent iteration of mapping and consensus calling. The total number of assembled viral reads was 5,600 (0.27% of the total reads). Despite the low proportion of viral sequence detected within the total data set, adequate coverage of the entire genome was obtained (average read depth of 58.16), with the exception of the highly conserved complementary untranslated regions (UTRs). The genomic termini sequence was obtained by using primers described previously (4).

The genetic organization of the E. isabellinus EBLV-1 genome was similar to those of other EBLV-1 genomes, with a complete genome size of 11,964 nucleotides. The coding region lengths were conserved; however, indels were observed in a number of intergenic regions. A 6-bp indel in the N-P intergenic region (1478 to 1483), which was previously identified within the EBLV-1 species (8), was observed. Interestingly, only the EBLV-1b full genome from E. serotinus from France had the 6-bp insertion; all other EBLV-1 genomes, including the E. isabellinus EBLV-1, did not. Two further indels were identified, (i) A^2420^ in the P-M region and (ii) C^5249^ in the G-L region. Both nucleotides were absent in the E. isabellinus EBLV-1 in comparison to the six EBLV-1 genomes available. It is unlikely that the observed indels have any biological influence; however, they support the independent evolution of the Iberian peninsula EBLV-1 viruses to other EBLV-1 viruses, due to geographical and host separation.

### Nucleotide sequence accession number. 

The complete genomic sequence of RV2416 (292R07) has been deposited in GenBank under the accession number KP241939.
